# Bioaugmentation failed to enhance oil bioremediation in three soil samples from three different continents

**DOI:** 10.1038/s41598-019-56099-2

**Published:** 2019-12-20

**Authors:** Samir S. Radwan, Dina M. Al-Mailem, Mayada K. Kansour

**Affiliations:** 1 Department of Biological Sciences, Faculty of Science, Kuwait University, P O Box 5969, Safat, 13060 Kuwait; 2 Present Address: Von Einem Str. 25, 48159 Münster, Germany

**Keywords:** Bacteriology, Soil microbiology, Microbial ecology

## Abstract

Soil samples from Kuwait, Lebanon, Egypt and Germany were polluted with 3% crude oil. Series of samples were left unbioaugmented, others were bioaugmented with Kuwaiti desert soil with a long history of oil pollution and still others with Kuwaiti marine biofouling material. In the samples from Kuwait, Egypt, and Germany, bioaugmentation did not enhance oil removal, whereas it did in the sample from Lebanon. Taxa from the desert-soil bioaugmented batches, but none of those from the biofouling-material bioaugmented ones, succeeded in colonizing the four studied soils. The dynamics of the hydrocarbonoclastic communities during bioremediation were monitored. Those communities differed in composition, not only according to the type of soil, but also for the same soil; at various phases of bioremediation. Although each soil seemed to have its characteristic microflora, they all were similar in harboring lower and higher actinomycetes and pseudomonads in addition to many other taxa. None of the taxa prevailed through all phases of bioremediation. The most powerful isolate in oil-removal; was *Rhodococcus erythropolis* (Germany), and the weakest was *Arthrobacter phenanthrenivorans* (Lebanon). The pure hydrocarbonoclastic isolates tolerated unusually high oil concentrations, up to 30%.

## Introduction

Increasing amounts of crude oil are being produced, processed and used worldwide as an energy source. According to earlier estimates, between 0.08 and 0.4% of the world’s oil is spilled contaminating the marine environment^1^. Given that the above estimates are becoming much higher, and also that the terrestrial and atmospheric ecosystems receive big shares of oil pollution, the spilled crude oil and its processed products globally represent a huge environmental hazard. Oil and oil products are known to be slowly biodegradable^2^ and many constituent hydrocarbons, especially the poly-aromatics, are toxic, carcinogenic or genotoxic^3,4^.

Chemical and physical remediation approaches are commonly not cost-effective^5^, and are not always environmentally safe. In contrast, bioremediation technology^6,7^ overcomes these disadvantages, making use of the biodegradation potential of naturally occurring hydrocarbonoclastic microorganisms in digesting oil and hydrocarbon pollutants.

For bioremediation, two distinct approaches are known. The first is bioaugmentation (also called seeding or inoculation). In this approach exogenous hydrocarbonoclastic microorganisms are introduced into the polluted site^8,9^. The second approach is biostimulation. It involves enhancing the hydrocarbonoclastic activities of microorganisms already inhabiting the polluted site by specific managements^6,10,11^. Thus, bioaugmentation but not biostimulation, involves addition of more gene pools to the already existing ones^12^, and the newly introduced microorganisms have to withstand the competition stress exerted by the native microorganisms^13^. Should this fail, the bioremediation activity would not be enhanced or might even be inhibited. In an earlier publication, our group showed that bioaugmented hydrocarbonoclastic *Arthrobacter* strains imported from Germany failed to colonize oil polluted Kuwaiti soils, whereas locally isolated *Arthrobacter* strains colonized them successfuly^14^.

A question that still needs to be answered is related to which microorganisms are best for bioaugmentation of oily environments. Studies in this field are numerous; single or mixed cultures of hydrocarbonoclastic microorganisms or natural products rich in them are usually used^15^. There are cocktails of hydrocarbonoclastic microorganisms available commercially^16,17^. In recent years, the term autochthonous bioaugmentation (ABA), coined by Ueno^18^, has become popular and in which only organisms indigenous to the polluted site are used for successful bioremediation^11,19,20^. Autochthonous inhabitants are those perfectly adapted to the environment and which therefore; contribute significantly to biochemical activities there^21^. Allochthonous inhabitants are transitionally present in the environment, which is not their natural habitat. Therefore, they are not used for bioaugmentation because they perform only limited biochemical activities, just enough for them to survive.

With these facts in mind, we collected soil samples from 3 different continents, polluted them with oil and bioaugmented them with local Kuwaiti materials rich in hydrocarbonoclastic microorganisms. Unbioaugmented samples were used as controls. Through 6 months, the fate of oil and the microbial population dynamics in these soils were monitored. We also studied the colonization capability of pure bacterial cultures that we isolated from the bioaugmentation materials in the treated soil samples as well as the oil-tolerance and oil-consumption by representative isolates. The objectives were to investigate the feasibility of bioaugmentation as a bioremediation approach and to deepen our understanding of the terms autochthonous and allochthonous bioaugmentation.

## Results

### Hydrocarbonoclastic microbial communities in the pristine soils and bioaugmentation materials

Table 1 presents the results of analysis of the hydrocarbonoclastic bacteria in the four studied pristine (no oil added) soil samples and the two bioaugmentation materials. The colony forming units (CFU), as counted on a solid mineral medium with oil vapor as a sole carbon and energy source^22^, ranged in numbers between 3 ± 0.1 × 10^5^ g^−1^ for the Egyptian sample and 18 ± 0.9 × 10^5^ g^−1^ for the Kuwaiti sample. The CFU numbers for the two studied bioaugmentation materials from Kuwait were 5 ± 0.2 × 10^5^ g^−1^ for the oily desert soil and 264 ± 14 × 10^5^ g^−1^ for the marine biofouling material.

**Table 1:** Hydrocarbonoclastic bacterial communities in the pristine soil samples and the bioaugmentation materials.

Sample	CFU × 10^5^ g^−1^	Constituent species	% of the total
**Soil**			
Kuwait	18 ± 0.9	*Rhodococcus jostii*	61.9
		*Streptomyces griseoflavus*	14.2
		*Streptomyces pluripotens*	12.5
		*Pseudomonas composti*	11.4
Lebanon	15 ± 0.7	*Sphingomonas kyeonggiensis*	53.7
		*Streptomyces bambusae*	31.7
		*Streptomyces racemochromogenes*	5.5
		*Rhodococcus globerulus*	2.8
		*Saccharothrix saharensis*	2.8
		*Saccharomonospora azurea*	2.1
		*Arthrobacter agilis*	1.4
Egypt	3 ± 0.1	*Nocardia neocaledoniensis*	35.7
		*Sphingomonas kyeonggiensis*	35.7
		*Streptomyces scopiformis*	17.9
		*Streptomyces bambusae*	10.7
Germany	13 ± 0.6	*Microbacterium ginsengiterrae*	35.7
		*Streptomyces bambusae*	31.0
		*Rhodococcus tukisamuensis*	11.9
		*Nocardia fluminea*	9.5
		*Psychrobacter muriicola*	7.1
		*Salinicoccus hispanicus*	4.0
		*Sphingomonas kyeonggiensis*	0.8
**Bioaugmentation materials**			
Oily desert soil	5 ± 0.2	*Pseudoxanthomonas japonensis*	66.0
		*Pseudomonas hunanensis*	20.7
		*Bosea massiliensis*	13.3
Marine biofouling material	264 ± 14	*Pontibaca methylaminivorans*	44.8
		*Planococcus maritimus*	28.1
		*Pseudoalteromonas undina*	27.0
		*Pseudoalteromonas atlantica*	0.1

Values are means of 3 replicate determinations ± standard deviation. Five representative isolates were made in each case.

Each of the studied four soil samples and the two bioaugmentation materials had its characteristic microbial-community composition. Thus, the pristine Kuwaiti-soil sample contained as predominant hydrocarbonoclastic bacteria *Rhodococcus jostii* and *Streptomyces griseoflavus*, the Lebanese sample, *Sphingomonas kyeonggiensis* and *Streptomyces bambusae*, the Egyptian sample, *Nocardia neocaledoniensis*, *Sphingomonas kyeonggiensis* and 2 *Streptomyces spp*. and the German sample, *Microbacterium ginsengiterrae* and *Streptomyces bambusae*. It is noteworthy that each of the four samples contained *Streptomyces* species as predominant hydrocarbonoclastic partner. *Sphingomonas kyeonggiensis*, one of the two predominant species in the Lebanese and Egyptian samples, occurred as a minor species in the German sample but was not detected in the Kuwaiti sample. Similarly, *Streptomyces bambusae*, one of the dominant species in the Lebanese and German samples occurred as a minor constituent in the Egyptian sample and was absent in the Kuwaiti sample. The bacterial communities in the two bioaugmentation materials were quite different in composition from one another and from the four soil samples studied.

### Oil bioremediation in unbioaugmented and bioaugmented soil samples

**Figure 1:**
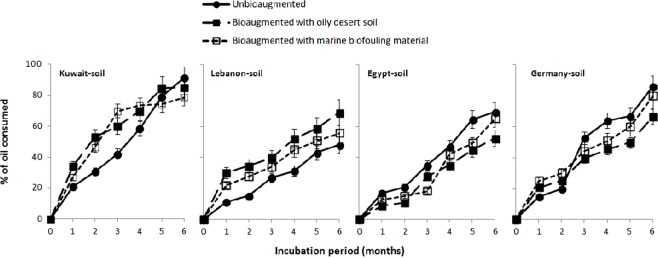
Crude-oil removal in unbioaugmented and bioaugmented four soil samples during bench-scale bioremediation. Each value was the mean of three parallel replicates.

Figure 1 shows that crude oil that had been mixed with the four soil samples was gradually biodegraded through the 6 months of bioremediation. The analysis of covariance (ANCOVA) showed that in all cases, time was a significant predictor of oil-consumption. In the Kuwaiti soil, the means of oil-consumption values were not significantly different between the unbioaugmented and bioaugmented samples while controlling the effect of the covariant (time). The same was true for the Egyptian soil. Only in the Lebanese soil, the means of oil-consumption in both bioaugmented samples were significantly different from those of the unbioaugmented control but the slops were not. For the German soil, bioaugmentation specially with biofouling material significantly reduced the oil-biodegradation values as compared with the unbioaugmented control. The P-values and F statistics etc, are available in the output in Table S1 in the Supplementary File. Available are also the corresponding ANCOVA plots. The plot was produced by R package “HH” (3.1.37) with the function *ancovaplot* (Fig. S1).

**Figure 2:**
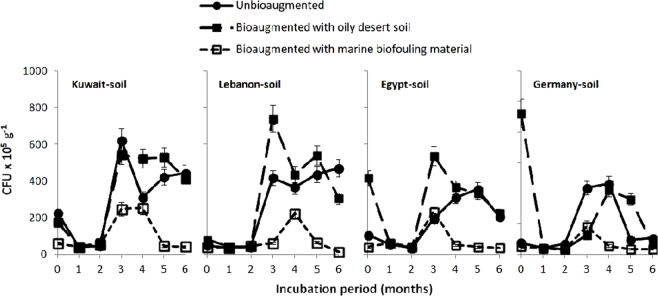
Changes in the numbers of CFUs of hydrocarbonoclastic bacteria in the four soil samples during bench-scale oil-bioremediation. Each value was the mean of five parallel replicates.

Figure 2 illustrates the changes in the numbers of CFU in the studied samples during bioremediation. Comparing the CFU numbers in the pristine soil samples without oil addition (in Table 1) with the numbers in the oil-treated, unbioaugmented batches at time zero (Fig. 2) reveals that the mere presence of oil instantaneously enhanced the hydrocarbonoclastic bacterial numbers by 18, 35, 3 and 6 fold in the Kuwaiti, Lebanese, Egyptian and German soils, respectively. The numbers in all the samples decreased significantly (ANOVA, n = 5, *P* < 0.05) during the first 2 months of bioremediation but increased significantly (ANOVA, n = 5, *P* < 0.05) in the third and fourth months chronologically with the maximum oil-removal rates (ANOVA, n = 5, *P* < 0.05). During the last 2 months of bioremediation the CFU numbers decreased significantly in all samples (ANOVA, n = 5, *P* < 0.05).

### Effects of specific treatments on whole bacterial community structures during bioremediation

**Figure 3:**
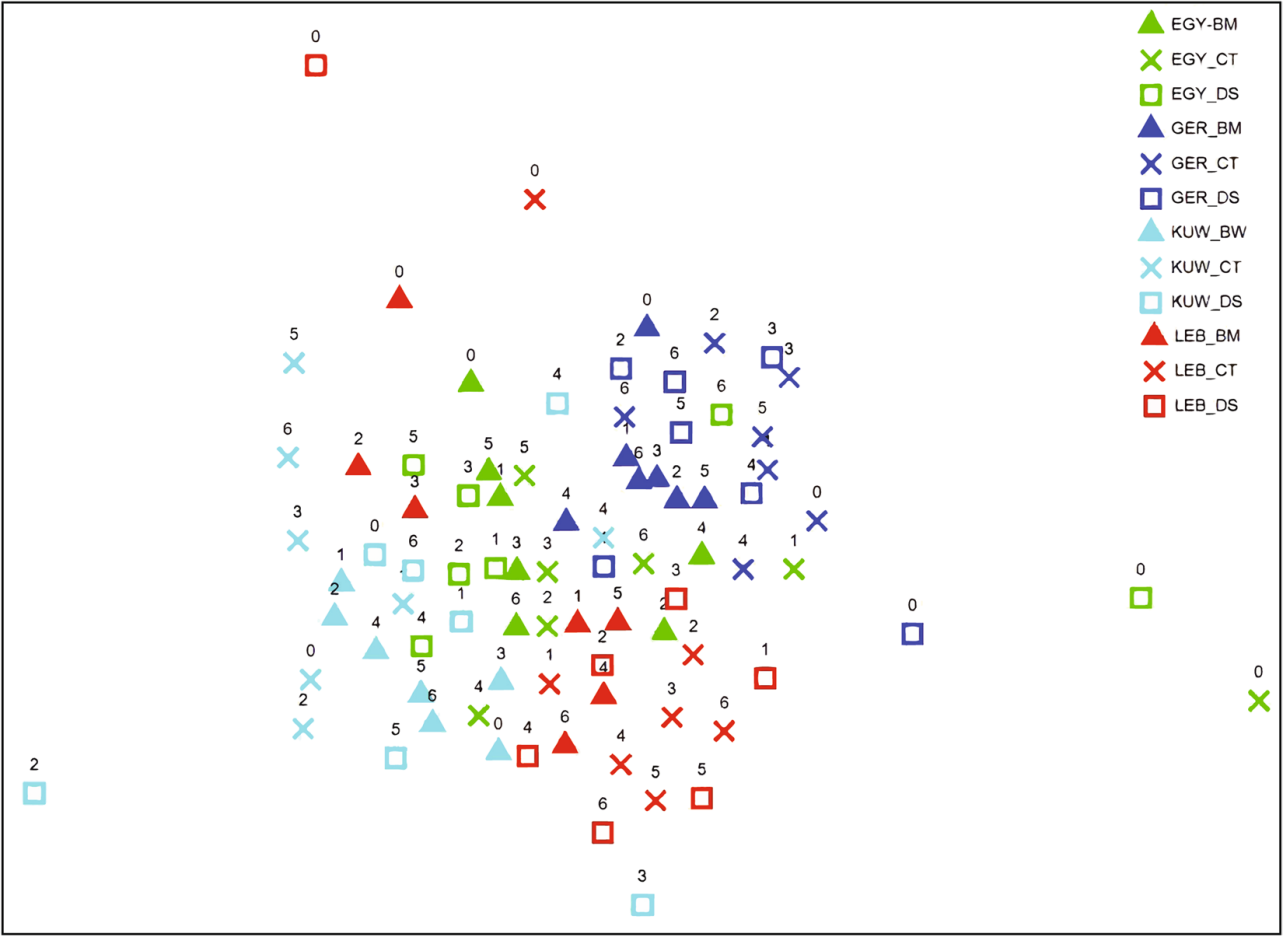
nMDS plot showing percentage similarities of the studied bacterial communities abundance. Colors represent countries of origin (light blue, Kuwait; red, Lebanon; green, Egypt; dark blue, Germany). Shapes represent treatments (X, control; square, Kuwait desert soil; triangle, biofouling material). Data was subjected to a Bray Curtis similarity matrix, MDS plot 2D stress 0.1.

Figure 3 presents a non-metric multidimensional scaling (nMDS) plot showing percentage similarities of the abundance of the bacterial communities. Table 2 summarizes the analysis of similarity results among the unbioaugmented control soils on one hand and soils bioaugmented with oily desert soil and biofouling material on the other hand. Across the entire dataset, there was some separation by location that was statistically significant (ANOSIM, R = 0.355, P = 0.001). There were no significant differences between control samples from any of the studied countries and those bioaugmented with oily desert soil, yet there was a weak but statistically significant difference with the soils bioaugmented with biofouling material in the German, Kuwaiti and Lebanese soils; with a weaker and less significant variation in the Egyptian soil. Table 2 includes the detailed analysis of similarities (ANOSIM).

**Table 2:** Analysis of similarity results between unbioaugmented controls and samples bioaugmented with oily desert soil and biofouling material.

Sample origin	Bioaugmented with	*R* value	Significance
Kuwait (control)	Oily desert soil	−0.071	0.79
	Bioaugmented material	0.328	0.006
Lebanon (control)	Oily desert soil	0.06	0.67
	Bioaugmented material	0.298	0.002
Egypt (control)	Oily desert soil	0.024	0.35
	Bioaugmented material	0.132	0.098
Germany (control)	Oily desert soil	−0.104	0.85
	Bioaugmented material	0.302	0.003

### Dynamics of bacterial species during bioremediation of the four studied soils

Figures S2–S5 in the Supplementary File show the composition of the hydrocarbonoclastic communities as well as their turnover in relation to their origin, bioaugmentation and time. Scanning the graphs in Figs. S2–S5 from the top to the bottom reveals the effects of time and from left to right, the effects of the bioaugmentation treatments. The effect of the microbial origin is revealed by comparing the 4 figures together. In this context, the differentiation between the native and inoculated strains could be concluded from Figs. S2–S5 by comparing the microbial identities in the bioaugmented and the unbioaugmented samples at time zero.

### Dynamics of bacteria in the kuwaiti soil

In the unbioaugmented batches of the Kuwaiti soil at time zero, the predominant bacteria were *Rhizobium alkalisoli* (strain, s.1) and, albeit to a less extent, *Pseudoxanthomonas japonensis* (s.3) (Fig. S2). One month later, *Sphingopyxis fribergensis* (s.21) took over the predominance together with *Pseudomonas aeruginosa* (s.14). None of the taxa was recorded at time zero. After 2 months, another group v.z. *Sagittula stellata* (s.36), *Pseudoxanthomonas japonensis* (s.3) and *Pseudoxanthomonas mexicana* (s.4) predominated; the second species was recorded at time zero. In months 3, 4, 5 and 6, during which most of the oil had been removed, *Pseudoxanthomonas japonensis* (s.3), *Xanthobacter flavus* (s.46), *Lacibacterium aquatile* (s.49) and *Bacillus thioparans* (s.28) predominated, respectively.

In the oily-soil bioaugmented batches, predominance patterns rather similar to those in the unbioaugmented batches prevailed at time zero and after 1, 2, 4 and 6 months. After 3 months, *Kocuria polaris* (s.40) predominated and after 5 months, *Pseudomonas mendocina* (s.15) and *Mycobacterium vanbaalenii* (s.33) prevailed. In the biofouling-material bioaugmented batches, there were similarities in the predominance patterns but only at time zero and after 5 and 6 months of bioremediation. Between months 1 and 4, species belonging to the genera *Pseudomonas*, *Sphingopyxis*, *Mycobacterium* and *Tistrella* predominated.

### Dynamics of bacteria in the lebanese soil

In the unbioaugmented sample at time zero, *Arthrobacter phenanthrenivorans* (s.1) and *A. ginsengisoli* (s.2) predominated (Fig. S3 in the Supplementary File). After one month, *Sphingobium quisquiliarum* (s.15), and after two months, the 3 species *Cellulomonas massiliensis* (s.38), *Actinotalea ferrariae* (s.36) and *Azospirillum doebereinerae* (s.19) prevailed. After 3 and 4 months, *Pseudomonas hunanensis* (s.32) took over the absolute predominance. After 5 months; *Actinotalea ferrariae* (s.36) and *Roseomonas aestuarii* (s.45), and after 6 months; *Pseudomonas benzenivorans* (s.34), were the most dominant strains.

The oily-desert soil bioaugmented batches also showed similar predominance patterns to those of the unbioaugmented batches. On the other hand, the marine-biofouling-material bioaugmented samples exhibited in most of the bioremediation phases quite different patterns of predominance (with the only exception of the 5-month batches). Thus, the marine species *Psychrobacter piscatorii* (s.8) and *Marinobacter adhaerens* (s.10) prevailed at time zero. After one month, *Actinotalea ferrariae* (s.36) predominated. After two, three and four months, *Georgenia daeguensis* (s.42), *Tessaracoccus oleiagri* (s.47) and *Dietzia papillomatosis* (s.41) prevailed, respectively. In the last months, 5 and 6, *Actinotalea ferrariae* (s.36) took over the predominance again. It is noteworthy that this latter strain was one of the prevailing taxa in the unbioaugmented and the oily-desert soil bioaugmented batches.

### Dynamics of bacteria in the egyptian soil

In the unbioaugmented batch at time zero, 3 taxa, *Arthrobacter flavus* (s.1), *Streptomyces lateritius* (s.7) and *Nocardioides luteus* (s.4) prevailed (Fig. S4 in the Supplementary File). One month later, *Paenibacillus lautus* (s.15) and *Pseudomonas knackmussii* (s.26) predominated. After two months, the 3 pseudomonads, *P. monteilii* (s.30), *P. benzenivorans* (s.31) and *P. knackmussii* (s.26) took over the predominance. At the third month, another 3 strains, *Mycobacterium vanbaalenii* (s.38), *Zavarzinia compransoris* (s.43) and *Streptomyces griseoflavus* (s.11) predominated. In month 4, *Mycobacterium vanbaalenii* (s.38) together with *Pseudomonas hunanensis* (s.32) also prevailed. In the last two months, *Zavarzinia compransoris* (s.43), together with *Pseudomonas spp*., predominated again.

The strains prevailing in the unbioaugmented batches also prevailed in the oily-desert soil bioaugmented batches: after one month (*Pseudomonas knackmussii*, s.26), four months (*Mycobacterium vanbaalenii*, s.38), five months (*Pseudomonas aeruginosa*, s.33) and six months (*Zavarzinia compransoris*, s.43). On the other hand, the marine-biofouling material bioaugmented batches showed quite different predominance patterns. Thus, *Arthrobacter phenanthrenivorans* (s.2), *Streptomyces leeuwenhoekii* (s.8), *Bacillus cavernae* (s.20) and *Psychrobacter pacificensis* (s.22) prevailed at time zero, *Pseudomonas aestusnigri* (s.29), *Algoriphagus olei* (s.36) and *Citreicella marina* (s.37) after one month, *Actinotalea ferrariae* (s.42), *Pseudomonas knackmussii* (s.26) and *Streptomyces atrovirens* (s.12) after two months, *Mycobacterium vanbaalenii* (s.38) and *Pseudomonas aeruginosa* (s.33) after three months, *Pseudomonas songnenensis* (s.27), *Gracilibacillus ureilyticus* (s.46) and *Marinobacter algicola* (s.24) after four months, *Kocuria dechangensis* (s.47), *Pseudomonas aeruginosa* (s.33) and *Streptomyces leeuwenhoekii* (s.8) after five months and *Bacillus oceanisediminis* (s.21), *Actinotalea ferrariae* (s.42) and *Streptomyces leeuwenhoekii* (s.8) after six months.

### Dynamics of bacteria in the german soil

In the unbioaugmented batch at time zero, *Rhodopseudomonas pseudopalustris* (s.1) and *Sphingomonas kyeonggiensis* (s.2) were predominant (Fig. S5 in the Supplementary File). One month later, *Xanthobacter flavus* (s.23), *Acidovorax facilis* (s.24) and *Rhodococcus erythropolis* (s.7) prevailed. After two months, *Xanthobacter flavus* (s.23) and *Nocardia fluminea* (s.3) predominated. After three months, *Rhodococcus erythropolis* (s.7) took over the predominance and it was replaced by *Zavarzinia compransoris* (s.32) after four months. After five months, this later species shared the predominance together with *Rhodococcus erythropolis* (s.7), *Streptomyces yaanensis* (s.13) and *Rhodococcus pedocola* (s.10).

The strains prevailing in the unbioaugmented batches also prevailed in the oily-desert soil bioaugmented batches at time zero and after 2, 3, 4 and 6 months. In the marine biofouling-material bioaugmented batches, the predominance pattern was quite different. *Rhodococcus erythropolis* (s.7) prevailed through the 6-month bioremediation period together with other species: *Microbacterium ginsengiterrae* (s.15) and *Paracoccus carotinifaciens* (s.20) at time zero, *Mycobacterium hackensackense* (s.18) and *Pseudomonas knackmussii* (s.27) after one month and *Microbacterium schleiferi* (s.16), *Mycobacterium hackensackense* (s.18) and *Gordonia amicalis* (s.29) after two months. From month 3 to month 6, *G.amicalis* (s.29) and *Rhodococcus erythropolis* (s.7) shared the predominance together with other strains: *Mycobacterium hackensackense* (s.18) and *Pseudomonas knackmussii* (s.27) in month 3, *Gordonia amicalis* (s.29) and *Zavarzinia compransoris* (s.32) in month 4, *Mycobacterium smegmatis* (s.19) and *Pseudomonas songnenensis* (s.28) in month 5 and *Mycobacterium hackensackense* (s.18) and *Pseudomonas knackmussii* (s.27) in month 6.

Figures S2–S5 in the Supplementary File reveal several bacterial species which were of common occurrence in more than one of the four soil samples studied. Thus, the Lebanese soil harbored 9 species which occurred in the Kuwaiti soil (strains 7, 12, 16, 17, 20, 33, 34, 43, 44). The Egyptian soil contained 21 species that existed also in the other soils (strains 8, 12, 14, 16, 17, 27, 33, 46, 48, 52, 53, 55, 61, 62, 70, 71, 74, 75, 79, 80, 88). The German soil accommodated 12 species also inhabiting the other 3 soils (strains 6, 43, 46, 61, 62, 71, 74, 75, 79, 88, 96, 105). Fig. S5 in the Supplementary File presents typical microscopic graphs of the 18 predominant isolates from the studied soil samples.

### Soil-colonization by bacteria inhabiting the bioaugmentation materials

Cells of the 3 pure cultures of *Pseudoxanthomonas japonensis*, *Pseudomonas hunanensis* and *Bosea massiliensis* (from the oily-desert soil of Kuwait) colonized the pristine soil samples effectively when inoculated as suspensions in sterile water. Thus, the total CFU of *P. japonensis* in the bioaugmented soil ranged in numbers between 0.6 and 1.3 × 10^6^g^−1^, of *P. hunanensis* exceeded 6 × 10^6^g^−1^ and of *B. massiliensis* between 2 and 4 × 10^6^g^−1^. On the other hand, cells of the three tested strains, *Planococcus maritimus*, *Pseudoalteromonas undina* and *Pontibaca methylaminivorans* (from the marine-biofouling material) completely failed to colonize the pristine soil samples.

### Oil-tolerance and –consumption by representative pure isolates

**Figure 4:**
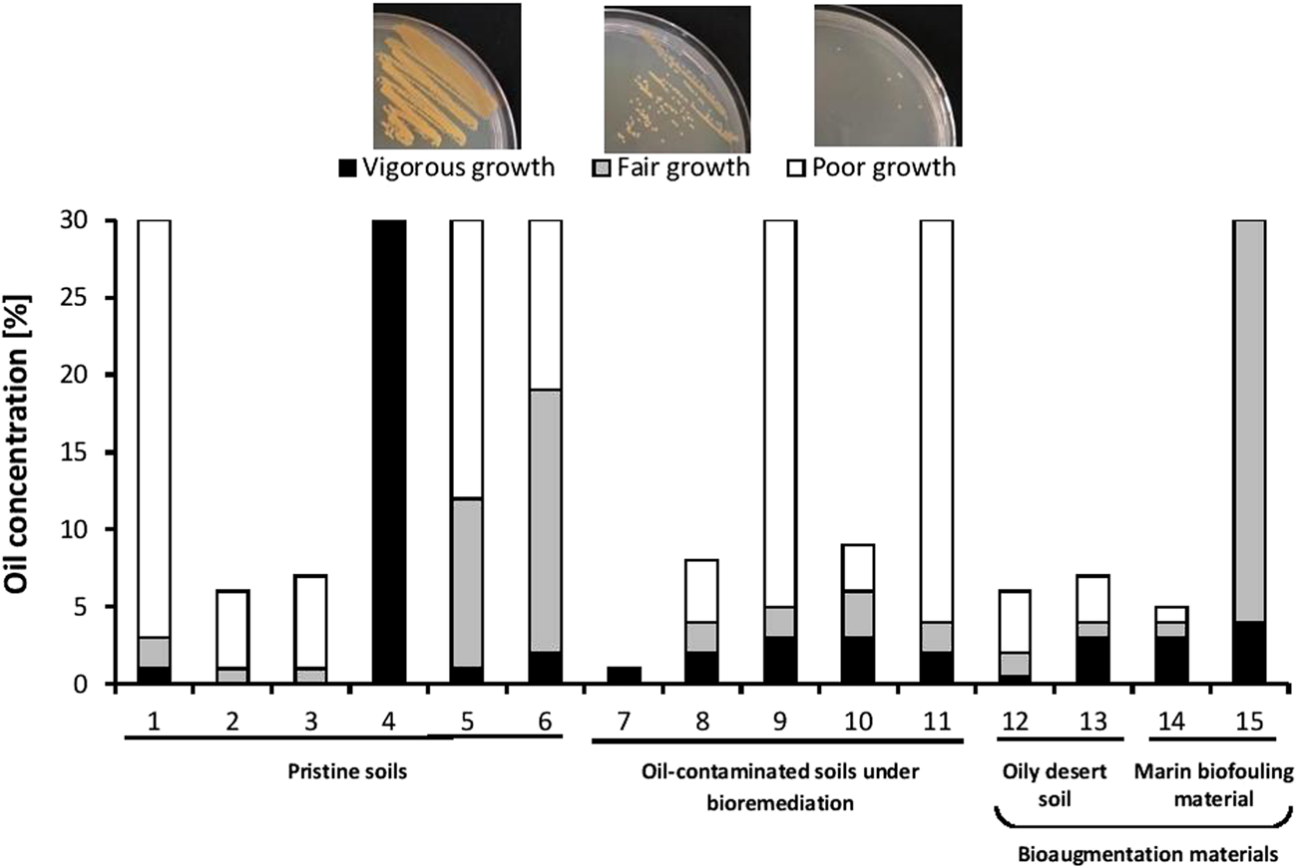
Highest oil concentrations tolerated by hydrocarbonoclastic bacterial isolates from the four studied soils (isolates 1–11) and the two bioaugmentation materials (isolates 12–15). 1, *Rhodococcus jostii*; 2, *Streptomyces griseoflavus*; 3, *Streptomyces bambusae*; 4, *Nocardia neocaledoniensis*; 5, *Sphingomonas kyeonggiensis*; 6, *Microbacterium ginsengiterrae*; 7, *Rhizobium alkalisoli*; 8, *Arthrobacter flavus*; 9, *Arthrobacter phenanthrenivorans*; 10, *Arthrobacter ginsengisoli*; 11, *Rhodopseudomonas pseudopalustris*; 12, *Pseudoxanthomonas japonensis*; 13, *Pseudomonas hunanensis*; 14, *Pontibaca methylaminivorans*; 15, *Planococcus maritimus*. Note that those organisms are more widely distributed in the studied samples than in the sources specified in the figure (for this see Figs. S2–S5).

On account of the extensive experimental setup, this study was performed on representative strains that had been isolated from the pristine and bioremediated soil samples and from the bioaugmentation materials. Figure 4 shows the highest crude-oil concentrations tolerated by the individual strains tested. *Rhizobium alkalisoli*, which predominated in the Kuwaiti soil batches at time zero, tolerated only up to 1%, w/v, oil. All the other strains had a much higher oil-tolerance potential. The bioaugmentation strains from the oily-desert soil (bioaugmentation material), *Pseudoxanthomonas japonensis* and *Pseudomonas hunanensis* (which occurred also in many of the soil sample batches, see Figs. S2–S5 in the Supplementary File) tolerated up to 6 and 7% oil, respectively, while *Pontibaca methylaminivorans* from the marine biofouling material tolerated up to 5% oil. Seven of the tested strains that were also widely distributed in the four studied soil samples tolerated up to 30% oil, the highest concentration tested in this experiment.

**Figure 5:**
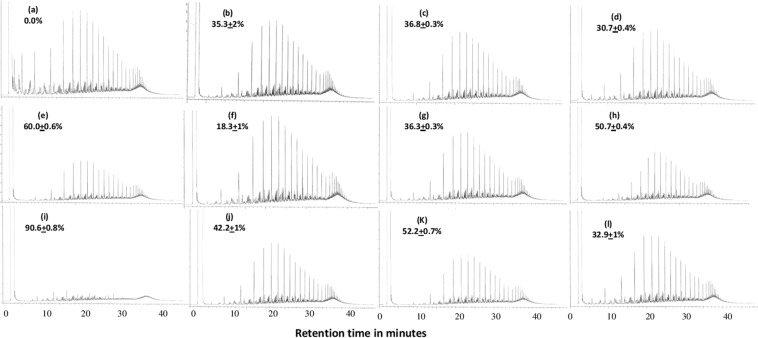
GLC profiles of crude oil recovered from cultures of pure isolates that grew for 10 days in a mineral medium containing 0.3% oil. (**a**) crude oil at time zero, (**b**) oil recovered from *Rhizobium alkalisoli* culture batch, (**c**) oil recovered from *Xanthobacter flavus* culture batch, (**d**) oil recovered from *Pseudomonas hunanensis* culture batch, (**e**) oil recovered from *Dietzia papillomatosis* culture batch, (**f**) oil recovered from *Arthrobacter phenanthrenivorans* culture batch, (**g**) oil recovered from *Arthrobacter flavus* culture batch, (**h**) oil recovered from *Mycobacterium vanbaalenii* culture batch, (**i**) oil recovered from *Rhodococcus erythropolis* culture batch, (**j**) oil recovered from *Microbacterium ginsengiterrae* culture batch, (**k**) oil recovered from *Zavarzinia compransoris* culture batch, (**l**) oil recovered from *Pseudoxanthomonas japonensis* culture batch. Note that those isolates are of wide distribution among the studied samples (see Figs. S2–S5). Values on the individual profiles are those of the oil-consumption values; they were means of 3 replicates, ± standard deviation values.

The typical GLC profiles of residual oil in cultures of 10 representative organisms that were also of wide distribution in the soil samples studied showed that the tested taxa varied in their potential for oil consumption (Fig. 5). The lowest potential of 18.3% consumption was that of *Arthrobacter phenanthrenivorans*, which inhabited Lebanese and Egyptian soils. The highest potential of 90.6% consumption was that of *Rhodococcus erythropolis* which predominated in the German soil sample at all stages of self-cleaning and bioremediation. The remaining strains consumed between 35 and 60% of the oil. The distribution of those tested strains in the various bioremediation batches is illustrated in Figs. S1–S4 in the Supplementary File.

## Discussion

In the literature, there is still a lot of contradiction regarding the effectiveness of bioaugmentation in combating environmental oil spills. For example, some authors reported that inoculating proper microorganisms into a site is not a guarantee of successful pollutant-removal^23^. On the other hand, hydrocarbons contaminating waste water were reported to be dramatically removed in response to water-bioaugmantation with a consortium of bacteria^12^. Inoculated microorganisms are known to face stiff competition with the native microflora during colonization of an environment, and consistent with this, intensive attempts in the last century to inoculate *Azotobacter* into soil as a substitute for chemical nitrogen fertilizers consistently failed^24^.

The bacterial communities in this work were studied by a culture-dependent approach on a medium with oil-vapor as a sole carbon source. Although it is known that this approach captures only a small part of the total community, it provided the valuable advantage of capturing only hydrocarbonoclastic microorganisms. A culture-independent method would not provide this advantage. Therefore, the isolates reported in this study should be looked at as predominant representatives of the hydrocarbonoclastic bacterial communities in the studied samples.

The main finding of the current study is that the native hydrocarbonoclastic bacterial communities in three soil samples from three different continents brought about equal or better oil-removal than when the samples had been bioaugmented with Kuwaiti desert soil with a long history of oil pollution (autochthonous bioaugmentation) or with a marine biofouling material (allochthonous bioaugmentation). A rather similar result was described in a recent study^25^. The only exception was the soil sample from Lebanon. Careful analysis of the results in Figs. 3 and S2–S5 in the Supplementary File shows that this sample exhibited some “uniqueness”, which makes it rather different from the other three samples. Thus, about 42% of its constituent hydrocarbonoclastic taxa (Fig. S3) did not show up in any of the other 3 samples whose “unique” taxa made only about 24, 8 and 24% of the total taxa in soils from Kuwait, Egypt and Germany, respectively. The “unique” isolates from Lebanon soil comprised many aquatic microorganisms, e.g. *Aquabacterium*, *Arcticibacter*, *Oceanobacillus* and even an *Escherichia sp*. (which is also hydrocarbonoclastic^26^). Furthermore, the Lebanese soil list was the only one free of *Bacillus spp*. and was poorer than the others in *Streptomyces spp*. and the nocardioforms (*Nocardia, Nocardiopsis* and *Rhodococcus*). Still more noteworthy is that the Lebanese soil harbored during bioremediation the hydrocarbonoclastic taxa with the weakest oil-removal potential. Thus, at time zero of bioremediation, there were two *Arthrobacter* species making together >96% of the total hydrocarbonoclastic bacteria in the unbioaugmented Lebanese soil (Fig. S3). The more dominant; *A. phenanthrenivorans* had the lowest oil-consumption potential of only 18.3% (Fig. 5). The corresponding organisms in the Kuwaiti, Egyptian and German samples were *Rhizobium alkalisoli* (with 35.3% consumption) and *Rhodopseudomonas pseudopalustris* (with no measured oil consumption). In month 3 (and later), chronologically with the most effective oil-consumption, the predominant strain in the Lebanese sample was *Pseudomonas hunanensis* (30.7% consumption), whereas in the Kuwaiti soil, *Pseudoxanthomonas japonensis* (32.9% consumption) and *Xanthobacter flavus* (36.8% consumption), in the Egyptian soil, *Mycobacterium vanbaalenii* (50.7% consumption) and *Zavarzinia compransoris* (52.2% consumption) and in the German soil, *Rhodococcus erythropolis* (90.6% consumption) and *Zavarzinia compransoris* (52.2% consumption) predominated. These facts indicate that oil removal by the native Lebanese soil microflora was weak and needed bioaugmentation to be enhanced. The fact that most of the isolates tolerated up to >30% crude oil means that severe oil-spills would be well tolerated by the hydrocarbonoclastic microbial communities native in the soil. Even weak growth would enable the strains to survive in environments supersaturated with oil. Within this context, this assay was performed in liquid cultures. The physic-chemical properties of oil in a sediment matrix (in soil) are different from those along the water-oil interface, yet the results recorded here may be useful in concluding that the isolates had some degree of oil-tolerance.

Another important point to be addressed is why oil-removal in many of the studied soil batches was more effective in the oily-desert soil bioaugmented samples than in those augmented with the marine-biofouling material. This is apparently because the former belongs to the so-called autochthonous bioaugmentation, whereas the latter is actually some sort of allochthonous bioaugmentation. The bioaugmented microorganisms in the former case were probably already adapted to the terrestrial physic-chemical parameters, whereas in the latter case the microorganisms were more suited to the marine physic-chemical parameters. This study provided experimental evidence for that. The first is the striking similarities in the bacterial community composition between the unbioaugmented and the oily-desert soil (but not the biofouling materials) bioaugmented batches during several phases of bioremediation. The second is that pure predominant species in the oily-desert soil, but not those in the marine biofouling material, succeeded in colonizing the studied soil samples effectively.

The last point to be addressed is why bacterial numbers in the studied soil samples increased instantaneously at time zero in response to the oil addition. Obviously, this increase was due to physical factors, not to the cell propagation which would have needed several days to occur. It is well known that the envelopes of many hydrocarbonoclastic bacterial species are hydrophobic^27,28^. In soil, their cells appear to be immobilized on hydrophilic cores, and the addition of oil probably results in their immediate release.

In conclusion, spilled-oil bioremediation in soil should, as a rule, depend on the indigenous microflora whose activities may be biostimulated by optimizing the prevailing physic-chemical parameters. The results of this study challenge bioaugmentation as a feasible approach for enhancing oil-bioremediation. The fact that the microbial communities vary dramatically in composition not only among the different soils, but also for the same soil at different phases of bioremediation, makes it impossible to decide which taxon (taxa) would be the most appropriate choice for bioaugmentation.

## Methods

### Soil samples and bioaugmentation materials

Pristine soil samples were collected in sterile containers from Kuwait-Asia (a desert soil sample from Al-Ahmadi area, 33 km south of Kuwait City), Lebanon-Asia (a garden soil sample from Al-Janoub area, 120 km south of Bierut), Egypt-Africa (a garden soil sample from a village, 120 km north of Cairo) and Germany-Europe (a sunflower-field soil sample at Münster/Westf, 850 km west-south of Berlin). Two environmental samples rich in hydrocarbonoclastic bacteria were collected as bioaugmentation materials. The first was a desert soil sample (from an oil field in Kadma, 40 km north of Kuwait City) and the second was a marine biofouling material (from Al-Khiran area, 70 km south of Kuwait City). Both samples had been used earlier in our laboratory in bioaugmentation experiments^29,30^.

### Experimental set up

For bench-scale bioremediation experiments, 100 g aliquots of the pristine soils were suspended in 100 portions of sterile water in conical flasks and mixed with 3 g portions of light Kuwaiti crude oil. The bioaugmentation materials were first homogenized in calculated volumes of sterile water, and equal homogenate volumes equivalent to 5 g of the material were inoculated into the flasks. The setup also included unbioaugmented flask. Three replicates were prepared throughout. The flasks were sealed and incubated under room conditions (about 27 °C). At time zero and monthly (up to 6 months), triplicate flasks were harvested for microbiological analysis and measurement of oil consumption.

### Microbiological analysis

For counting hydrocarbonoclastic bacteria, the plating method on a mineral medium with oil vapor as a sole carbon source was used^22^. One gram of soil was suspended in 99 ml sterile water giving the stock suspension (10^−2^) from which series of dilutions were prepared. Aliquots, 0.1 ml of each dilution, were spread on the solid mineral medium in Petri-dishes and crude oil vapor was made available as a sole carbon and energy source from 3 ml oil-impregnated filter papers fixed in the dish lids. Dishes were sealed with cello-tape and incubated at 30 °C for 12 days. Five parallel plates were prepared for every dilution. The colony forming units (CFU’s) were counted. Strains in the pooled replicate plates were categorized according to their colony and cell morphologies and were counted; representative colonies were isolated, purified and maintained on the above medium containing 1% crude oil. The isolates were subcultured every other week.

For characterization of the isolates, their 16S rRNA-genes were sequenced and the sequences compared with those of type strains in GenBank. To extract the total genomic DNA, 300 mg of the fresh 36-hour bacterial biomass was homogenized in 100 µl of PrepMan Ultra Sample Preparation Reagent (Applied Biosystems, USA) and 200 µl molecular water (Sigma, UK). The mixture was incubated in a water bath for 10 min at 100 °C, cooled for 2 min and then centrifuged at 14,000 × *g* for 3 min to collect the DNA-containing supernatant. The 16S rRNA-genes were amplified by the polymerase chain reaction (PCR). The reaction mixture contained puReTaq Ready-To-Go PCR Beads (Amersham Biosciences, UK), 1 µl (25 ng) of DNA template, and 1 µl each of the universal primer combinations GM5F (50-CCTACGGGAGGCAGCAG-30) and 907 R (50-CCGTCAATTCMTTTGAGTTT-30)^31^. The reaction volume was made up to 25 µl with molecular water. Amplification was done in a Veriti Thermal Cycler (Applied Biosystems, USA) by touch-down PCR in which the initial denaturation was at 95 °C for 5 min, and the annealing temperature started at 65 °C and decreased by 1 °C every cycle to 55 °C; 15 additional cycles were carried out at this temperature. The PCR products were purified using a QIA quick PCR purification kit (Qiagen, USA) to remove the Taq polymerase, primers and dNTPs. Partial sequencing of the 16S rRNA-gene was done using a BigDye version Terminator Kit (Applied Biosystems, USA); 20 ng of the DNA template was added to 2 µl of a Big Dye v 3.1 terminator and 2 µl of Big Dye Terminator v 1.1, v 3.1 5X sequencing buffer; l µl of either 907 R or GM5F was added to the mixture, and the final volume was brought up to 10 µl with molecular water. Labelling was completed in a Veriti Thermal Cycler (Applied Biosystems, USA) using one cycle of 96 °C for l min, then 25 cycles of l min at 96 °C, 5 s at 50 °C and 4 min at 60 °C. The pure template DNA samples were processed in a 3130xl genetic analyzer (Applied Biosystems, USA). Sequencing analysis version 5.2 software (Applied Biosystems, USA) was used to analyze the results. Sequences were subjected to basic local alignment search tool analysis with the National Center for Biotechnology Information (NCBI; Bethesda, MD, USA) GenBank database^32^.

The 149 hydrocarbonoclastic bacterial strains that had been isolated in this study are listed in Table S2 (Supplementary File), which includes data related to the sequencing of their 16S rDNA and their accession numbers in GenBank. The Table also shows that the sequence similarities of all strains to those of the type strains were between 99 and 100%.

To visualize, compare and interpret the bacterial community structures of the different soil samples, the relative abundances of the communities were analyzed using Primer 6 software^33^. In Primer 6, a resemblance matrix was created based on the Bray Curtis similarity index from the relative abundance^34^. Nonmetric multidimensional scaling (MDS) was performed on the resemblance matrix, which displays relative similarities between communities as distance (i.e. the closer two samples are the more similar the community). 2D MDS plots with a stress value of less than 0.2 were used as they were considered to have accurate information. Analysis of Similarity (ANOSIM) analyses were performed on the resemblance matrix to test specific hypotheses formed from interpretation of MDS plots.

### Measurement of oil-consumption

Triplicate cultures were harvested at time zero and monthly for 6 months. The residual oil was recovered by extraction with three successive portions of 15 ml pentane. The volume of the combined extract was made to 50 ml with pentane and 1 µl was analyzed by gas liquid chromatography (GLC). Hydrocarbon consumption was expressed in terms of percentage of total peak-area reduction based on the peak areas of the controls (time-zero flasks). The GLC was done using a Chrompack (NJ, USA) CP-9000 instrument equipped with a FID, a WCOT fused silica CP-Sil capillary column, and a temperature program of 45–310 °C, raising the temperature at a rate of 10 °C min^−1^.

### Colonization of soils with bioaugmented pure isolates

Pristine soil portions, 50 g, were wetted with 50 ml aliquots of sterile water and each was inoculated with 1 ml of a common inoculum of the tested organism containing about 10^9^ cells. The cultures were incubated at 30 °C for 5 days and the constituent microorganisms were plated as described above. Pure cultures of the tested organisms were also plated. Colonies in the plates of soil suspensions that were identical with those of the plated pure cultures in microscopic and staining characteristics were recognized and counted.

### Highest oil concentration tolerated by the isolates

The tested organisms were inoculated in mineral medium^22^ aliquots containing increasing amounts of 0.5 up to 30%, w/v, crude oil. The cultures were electrically shaken at; 120 rpm for 24 h at 30 °C. One loopful of the culture was streaked on conventional nutrient agar to test for cell viability. After incubation for 5 days at 30 °C, cultures were examined for growth and; vigor, and the highest tolerated oil concentration was recorded.

### Statistical analysis

Triplicate determinations for each analysis were done and the mean values, ± standard deviation values, were calculated using Microsoft Excel 2007. Statistical Package for Social Sciences, version 12, was used to assess the degree of significance. The analysis of variance (ANOVA) was used to differentiate between the means of the tested parameters. An analysis of covariance (ANCOVA) on individual sites with time as the covariate, oil-consumption as the dependent variable, and treatment as the categorical dependent variable was conducted by using R statistical environment (3.6.1) adopting the general formula aov (Oil_consumption ~ Time * Treratment, dat = Site_data). The results of the nMDS study were subjected to Bray Curtis similarity matrix, MDS plot 2D stress 0.1.

## Supplementary information

Table S1, Table S2, Figure S1 - Figure S6

## References

[1] National Research Council (NRC). Using Oil Spill Dispersants on the Sea. National Academy Press, Washington, DC (1989).

[2] Andreoni V, Gianfreda L (2007). Bioremediation and monitoring of aromatic-polluted habitats. Appl. Microbiol. Biotech..

[3] Sverdrup LE, Nielsen T, Krogh PH (2002). Soil ecotoxicity of polycyclic aromatic hydrocarbons in relation to soil sorption, lipophilicity, and water solubility. Environ. Sci. Technol..

[4] Gu SH, Kralovec AC, Christensen ER, Van Camp RP (2003). Source apportionment of PAHs in dated sediments from the Black River. Water Resour.

[5] Rosenberg E (1993). Exploiting microbial growth on hydrocarbons–new markets. Trends. Biotechnol..

[6] Atlas, R. M. & Bartha, R. Microbial Ecology: Fundamentals and Applications, 4th edition. Benjamin/Cummings Publishing Company Inc., Canada (1998).

[7] Piskonen R, Itävaara M (2004). Evaluation of chemical pretreatment of contaminated soil for improved PAH bioremediation. Appl. Microbiol. Biotechnol..

[8] Van Limbergen H, Top EM, Verstraete W (1998). Bioaugmentation in activated sludge: Current features and future perspectives. Appl. Microbiol. Biotechnol..

[9] Kuiper I, Lagendijk EL, Bloemberg GV, Lugtenberg JJ (2004). Rhizoremediation: a beneficial plant-microbe interaction. Mal. Plant. Microb. In..

[10] Al-Mailem DM, Al-Deieg M, Eliyas M, Radwan SS (2017). Biostimulation of indigenous microorganisms for bioremediation of oily hypersaline microcosms from the Arabian Gulf Kuwaiti coasts. J. Environ. Manage..

[11] Nikolopoulou M, Pasadakis N, Kalogerakis N (2013). Evaluation of autochthonous bioaugmentation and biostimulation during microcosm-simulated oil spills. Mar. Pollut. Bull..

[12] Domde P, Kapley A, Purohit HJ (2007). Impact of bioaugmentation with a consortium of bacteria on the remediation of wastewater-containing hydrocarbons. Enviro. Sci. Pollut. Res..

[13] Radwan SS (1990). Gulf oil spill. Nature.

[14] Radwan SS, Sorkhoh NA, El-Nemr IM, El-Desouky AF (1997). A feasibility study on seeding as a bioremidiation practice for the oily Kuwaiti desert. J. Appl. Microbiol..

[15] Dashti N, Ali N, Khanafer M, Radwan S (2017). Oil uptake by plant-based sorbents and its biodegradation by their naturally associated microorganisms. Environ. Poll..

[16] Applied Bioremediation Association. Case History Compendium. Applied Biotreatment association, Washington DC (1989).

[17] Applied Biotreatment Association. The Role of Biotreatment of oil spill. Applied Biotreatment Association, Washington DC (1990).

[18] Ueno A, Ito Y, Yumoto I, Okuyama H (2007). Isolation and characterization of bacteria from soil contaminated with diesel oil and the possible use of these in autochthonous bioaugmentation. World J. Microbiol. Biotechnol..

[19] Hosakawa R, Nagai M, Morikawa M, Okayama H (2009). Autochthonous bioaugmentation and its possible application to oil spills. World. J. Microbiol. Biotechnol..

[20] DiGregorio S, Castglione MR, Gentini A, Lorenzi R (2015). Biostimulation of the autochthonous bacterial community and bioaugmentation of selected bacterial strains for the depletion of polycyclic aromatic hydrocarbons in a historically contaminated soil. Geophys. Res. Abstracts..

[21] Winogradsky S (1924). Sur la microflora autochtone de la terre arable. Comtes redus hebdomadaires des séances de l’ Academie des Sciences (Paris). D.

[22] Sorkhoh NA, Ghannoum MA, Ibrahim AS, Stretton RJ, Radwan SS (1990). Crude oil and hydrocarbon degrading strains of Rhodococcus rhodochrous isolated from soil and marine environments in Kuwait. Environ. Pollut..

[23] El Fantroussi S, Agathos SN (2005). Is bioaugmentation a feasible strategy for pollutant removal and site remediation?. Current. Opin. Microbiol..

[24] Alexander, M. Introduction to soil microbiology. New York: Wiley. (1961).

[25] Fodelianakis S (2015). Allochthonous bioaugmentation in *ex situ* treatment of crude oil-polluted sediments in the presence of an effective degrading indigenous microbiome. J. Hazard. Mater..

[26] Khanafer Majida, Al-Awadhi Husain, Radwan Samir (2017). Coliform Bacteria for Bioremediation of Waste Hydrocarbons. BioMed Research International.

[27] Van Loosdrecht M, Lyklema J, Norde W, Schraa G, Zehnder A (1987). The Role of Bacterial Cell Wall Hydrophobicity in Adhesion. App. Environ. Microb..

[28] Castellanos T, Ascencio F, Bashan Y (1997). Cell-surface hydrophobicity and cell-surface charge of Azospirillum spp. FEMS. Microb. Ecol..

[29] Dashti Narjes, Ali Nedaa, Salamah Samar, Khanafer Majida, Al‐Shamy Ghada, Al‐Awadhi Husain, Radwan Samir S. (2018). Culture‐independent analysis of hydrocarbonoclastic bacterial communities in environmental samples during oil‐bioremediation. MicrobiologyOpen.

[30] Ali Nidaa, Dashti Narjes, Salamah Samar, Sorkhoh Naser, Al-Awadhi Husain, Radwan Samir (2016). Dynamics of bacterial populations during bench-scale bioremediation of oily seawater and desert soil bioaugmented with coastal microbial mats. Microbial Biotechnology.

[31] Santegoeds CM, Ferdelman TG, Muyzer G, Beer D (1998). Structural and functional dynamics of sulfate-reduction populations in bacterial biofilms. Appl. Enviro. Micorbiol..

[32] Altschul SF (1997). Gapped BLAST and PSI-BLAST: a new generation of protein database search programs. Nucleic. Acids. Res..

[33] Clarke, K. R. & Gorley, R. N. *PRIMER v6: User Manual/Tutorial*. PRIMER-E (2006).

[34] Bray RJ, Curtis JT (1957). An Ordination of the Upland Forest Communities of Southern Wisconsin. Ecological Monographs.

