# Supplementary material for: Bioaugmentation failed to enhance oil bioremediation in three soil samples from three different continents
Source: Sci Rep. 2019 Dec 20;9:19508. doi: 10.1038/s41598-019-56099-2 (PMC6925256; doi:10.1038/s41598-019-56099-2)
Supplement: Supplementary file 1 — Table S1, Table S2, Figure S1 - Figure S6 [file 41598_2019_56099_MOESM1_ESM.docx]

Manuscript title: Bioaugmentation failed to enhance oil bioremediation in three soil samples from three different continents

Authors: Samir S. Radwan, Dina M. Al-Mailem & Mayada K. Kansour

**Supplementary material**

**Table S1**  Significance data (P-values, F statistic etc.)

The Kuwaiti soil

> summary(aov(Oil_consumption ~ Time * Treatment, dat= oil[oil$Site =="Kuwait",]))

              Df Sum Sq Mean Sq F value   Pr(>F)

Time            1  15166   15166 132.467 7.63e-09 ***

Treatment       2    379     189   1.654    0.224

Time:Treatment  2     64      32   0.280    0.759

Residuals      15   1717     114

The Lebanese soil

> summary(aov(Oil_consumption ~ Time * Treatment, dat= oil[oil$Site =="Lebanon",]))

               Df Sum Sq Mean Sq F value   Pr(>F)

Time            1   6417    6417 168.314 1.48e-09 ***

Treatment       2    920     460  12.063 0.000754 ***

Time:Treatment  2     76      38   0.999 0.391313

Residuals      15    572      38

The Egyptian soil

> summary(aov(Oil_consumption ~ Time * Treatment, dat= oil[oil$Site =="Egypt",]))

               Df Sum Sq Mean Sq F value   Pr(>F)

Time            1   9564    9564 292.221 3.03e-11 ***

Treatment       2     75      37   1.141    0.346

Time:Treatment  2    153      77   2.340    0.130

Residuals      15    491      33

The German soil

> summary(aov(Oil_consumption ~ Time * Treatment, dat= oil[oil$Site =="Germany",]))

               Df Sum Sq Mean Sq F value   Pr(>F)

Time            1  12437   12437 497.260 6.48e-13 ***

Treatment       2    296     148   5.927   0.0127 *

Time:Treatment  2    252     126   5.046   0.0211 *

Residuals      15    375      25

Signif. codes:  0 ‘***’ 0.001 ‘**’ 0.01 ‘*’ 0.05 ‘.’ 0.1 ‘ ’ 1

**Table S2** Information related to 16S rRNA gene sequencing of the microbial isolates from the four studied soil samples

| **Isolate no.** | **Total bases** | **Subdivision** | **Nearest genebank match** | **Similarity %** | **Bases compared** | **Genebank accession no.** |
| --- | --- | --- | --- | --- | --- | --- |
| 1 | 555 | Betaproteobacteria | *Achromobacter spanius* | 100 | 555/555 | MH586569 |
| 2 | 566 | Betaproteobacteria | *Acidovorax facilis* | 100 | 566/566 | MH586570 |
| 3 | 549 | Actinobacteria | *Actinotalea ferrariae* | 100 | 549/549 | MH586571 |
| 4 | 535 | Actinobacteria | *Agrococcus citreus* | 100 | 535/535 | MH586572 |
| 5 | 541 | Actinobacteria | *Agromyces aurantiacus* | 99 | 549/553 | MH586573 |
| 6 | 508 | Actinobacteria | *Agromyces indicus* | 99 | 514/517 | MH586574 |
| 7 | 525 | Actinobacteria | *Agromyces mediolanus* | 100 | 525/525 | MH586575 |
| 8 | 544 | Bacteroidetes | *Algoriphagus olei* | 100 | 544/544 | MH586576 |
| 9 | 551 | Betaproteobacteria | *Aquabacterium olei* | 99 | 553/554 | MH586577 |
| 10 | 559 | Sphingobacteriia | *Arcticibacter pallidicorallinus* | 100 | 559/559 | MH586578 |
| 11 | 534 | Actinobacteria | *Arthrobacter agilis* | 100 | 534/534 | MH586579 |
| 12 | 528 | Actinobacteria | *Arthrobacter flavus* | 100 | 528/528 | MH586580 |
| 13 | 528 | Actinobacteria | *Arthrobacter ginsengisoli* | 100 | 528/528 | MH586581 |
| 14 | 537 | Actinobacteria | *Arthrobacter phenanthrenivorans* | 100 | 537/537 | MH586582 |
| 15 | 554 | Betaproteobacteria | *Azospira oryzae* | 100 | 554/554 | MH586583 |
| 16 | 527 | Alphaproteobacteria | *Azospirillum brasilense* | 100 | 527/527 | MH586584 |
| 17 | 488 | Proteobacteria | *Azospirillum doebereinerae* | 99 | 498/503 | MH586585 |
| 18 | 481 | Alphaproteobacteria | *Azospirillum oryzae* | 100 | 481/481 | MH586586 |
| 19 | 544 | Bacilli | *Bacillus aryabhattai* | 99 | 548/550 | MH586587 |
| 20 | 411 | Bacilli | *Bacillus cavernae* | 99 | 413/414 | MH586588 |
| 21 | 566 | Bacilli | *Bacillus hwajinpoensis* | 100 | 566/566 | MH586589 |
| 22 | 547 | Bacilli | *Bacillus oceanisediminis* | 100 | 547/547 | MH586590 |
| 23 | 548 | Bacilli | *Bacillus thioparans* | 100 | 548/548 | MH586591 |
| 24 | 530 | Alphaproteobacteria | *Bosea massiliensis* | 99 | 532/533 | MH586592 |
| 25 | 515 | Actinobacteria | *Cellulomonas massiliensis* | 100 | 515/515 | MH586593 |
| 26 | 540 | Alphaproteobacteri | *Citreicella marina* | 100 | 540/540 | MH586594 |
| 27 | 558 | Betaproteobacteria | *Delftia tsuruhatensis* | 100 | 558/558 | MH586595 |
| 28 | 537 | Actinobacteria | *Dietzia maris* | 100 | 537/537 | MH586596 |
| 29 | 539 | Actinobacteria | *Dietzia natronolimnaea* | 100 | 539/539 | MH586597 |
| 30 | 522 | Actinobacteria | *Dietzia papillomatosis* | 99 | 532/536 | MH586598 |
| 31 | 527 | Alphaproteobacteria | *Ensifer adhaerens* | 100 | 527/527 | MH586599 |
| 32 | 553 | Gammaproteobacteria | *Escherichia fergusonii* | 100 | 553/553 | MH586600 |
| 33 | 525 | Bacilli | *Fictibacillus halophilus* | 100 | 525/525 | MH586601 |
| 34 | 523 | Actinobacteria | *Georgenia daeguensis* | 99 | 525/526 | MH586602 |
| 35 | 549 | Actinobacteria | *Gordonia amicalis* | 100 | 549/549 | MH586603 |
| 36 | 515 | Actinobacteria | *Gordonia hankookensis* | 100 | 515/515 | MH586604 |
| 37 | 528 | Actinobacteria | *Gordonia terrae* | 100 | 528/528 | MH586605 |
| 38 | 549 | Bacilli | *Gracilibacillus ureilyticus* | 100 | 549/549 | MH586606 |
| 39 | 528 | Actinobacteria | *Janibacter hoylei* | 100 | 528/528 | MH586607 |
| 40 | 527 | Proteobacteria | *Kaistia terrae* | 100 | 527/527 | MH586608 |
| 41 | 420 | Actinobacteria | *Kocuria dechangensis* | 99 | 430/435 | MH586609 |
| 42 | 489 | Actinobacteria | *Kocuria polaris* | 100 | 489/489 | MH586610 |
| 43 | 521 | Alphaproteobacteria | *Lacibacterium aquatile* | 99 | 527/530 | MH586611 |
| 44 | 530 | Actinobacteria | *Lentzea flaviverrucosa* | 100 | 530/530 | MH586612 |
| 45 | 559 | Gammaproteobacteria | *Marinobacter adhaerens* | 100 | 559/559 | MH586613 |
| 46 | 553 | Gammaproteobacteria | *Marinobacter algicola* | 100 | 553/553 | MH586614 |
| 47 | 526 | Gammaproteobacteria | *Marinobacter salarius* | 99 | 538/544 | MH586615 |
| 48 | 551 | Betaproteobacteria | *Massilia varians* | 100 | 551/551 | MH586616 |
| 49 | 535 | Actinobacteria | *Microbacterium arthrosphaerae* | 100 | 535/535 | MH586617 |
| 50 | 537 | Actinobacteria | *Microbacterium ginsengiterrae* | 100 | 537/537 | MH586618 |
| 51 | 529 | Actinobacteria | *Microbacterium schleiferi* | 100 | 529/529 | MH586619 |
| 52 | 535 | Actinobacteria | *Micrococcus aloeverae* | 100 | 535/535 | MH586620 |
| 53 | 518 | Actinobacteria | *Mycobacterium arceuilense* | 99 | 520/521 | MH586621 |
| 54 | 554 | Actinobacteria | *Mycobacterium bacteremicum* | 100 | 554/554 | MH586622 |
| 55 | 542 | Actinobacteria | *Mycobacterium hackensackense* | 100 | 542/542 | MH586623 |
| 56 | 536 | Actinobacteria | *Mycobacterium iranicum* | 100 | 536/536 | MH586624 |
| 57 | 504 | Actinobacteria | *Mycobacterium psychrotolerans* | 99 | 508/510 | MH586625 |
| 58 | 418 | Actinobacteria | *Mycobacterium sediminis* | 100 | 418/418 | MH586626 |
| 59 | 534 | Actinobacteria | *Mycobacterium smegmatis* | 99 | 536/537 | MH586627 |
| 60 | 537 | Actinobacteria | *Mycobacterium vanbaalenii* | 100 | 537/537 | MH586628 |
| 61 | 546 | Gammaproteobacteria | *Nevskia aquatilis* | 100 | 546/546 | MH586629 |
| 62 | 536 | Actinobacteria | *Nocardia fluminea* | 100 | 536/536 | MH586630 |
| 63 | 543 | Actinobacteria | *Nocardia iowensis* | 100 | 543/543 | MH586631 |
| 64 | 511 | Actinobacteria | *Nocardia lijiangensis* | 100 | 511/511 | MH586632 |
| 65 | 539 | Actinobacteria | *Nocardia neocaledoniensis* | 100 | 539/539 | MH586633 |
| 66 | 521 | Actinobacteria | *Nocardia testacea* | 100 | 521/521 | MH586634 |
| 67 | 499 | Actinobacteria | *Nocardioides luteus* | 100 | 499/499 | MH586635 |
| 68 | 473 | Bacilli | *Oceanobacillus aidingensis* | 99 | 476/477 | MH586636 |
| 69 | 527 | Alphaproteobacteria | *Ochrobactrum tritici* | 100 | 527/527 | MH586637 |
| 70 | 567 | Bacilli | *Paenibacillus lautus* | 100 | 567/567 | MH586638 |
| 71 | 527 | Bacilli | *Paenibacillus silvae* | 99 | 529/530 | MH586639 |
| 72 | 524 | Alphaproteobacteria | *Paracoccus carotinifaciens* | 99 | 526/527 | MH586640 |
| 73 | 473 | Actinobacteria | *Phycicoccus dokdonensis* | 100 | 473/473 | MH586641 |
| 74 | 545 | Bacilli | *Planococcus maritimus* | 100 | 545/545 | MH586642 |
| 75 | 535 | Bacilli | *Planomicrobium glaciei* | 100 | 535/535 | MH586643 |
| 76 | 505 | Alphaproteobacteria | *Pontibaca methylaminivorans* | 100 | 505/505 | MH586644 |
| 77 | 539 | Gammaproteobacteria | *Pseudoalteromonas atlantica* | 100 | 539/539 | MH586645 |
| 78 | 540 | Gammaproteobacteria | *Pseudoalteromonas undina* | 100 | 540/540 | MH586646 |
| 79 | 573 | Gammaproteobacteria | *Pseudomonas aeruginosa* | 100 | 573/573 | MH586647 |
| 80 | 567 | Gammaproteobacteria | *Pseudomonas aestusnigri* | 100 | 567/567 | MH586648 |
| 81 | 470 | Gammaproteobacteria | *Pseudomonas alcaligenes* | 100 | 472/472 | MH586649 |
| 82 | 515 | Gammaproteobacteria | *Pseudomonas balearica* | 99 | 523/527 | MH586650 |
| 83 | 555 | Gammaproteobacteria | *Pseudomonas benzenivorans* | 100 | 555/555 | MH586651 |
| 84 | 547 | Gammaproteobacteria | *Pseudomonas composti* | 99 | 551/553 | MH586652 |
| 85 | 556 | Gammaproteobacteria | *Pseudomonas hunanensis* | 100 | 556/556 | MH586653 |
| 86 | 599 | Gammaproteobacteria | *Pseudomonas knackmussii* | 100 | 599/599 | MH586654 |
| 87 | 546 | Gammaproteobacteria | *Pseudomonas linyingensis* | 100 | 546/546 | MH586655 |
| 88 | 548 | Gammaproteobacteria | *Pseudomonas mendocina* | 100 | 548/548 | MH586656 |
| 89 | 436 | Gammaproteobacteria | *Pseudomonas monteilii* | 99 | 464/470 | MH586657 |
| 90 | 573 | Gammaproteobacteria | *Pseudomonas plecoglossicida* | 100 | 573/573 | MH586658 |
| 91 | 566 | Gammaproteobacteria | *Pseudomonas prosekii* | 100 | 566/566 | MH586659 |
| 92 | 552 | Gammaproteobacteria | *Pseudomonas resinovorans* | 100 | 552/552 | MH586660 |
| 93 | 555 | Gammaproteobacteria | *Pseudomonas songnenensis* | 100 | 555/555 | MH586661 |
| 94 | 463 | Actinobacteria | *Pseudonocardia alni* | 100 | 463/463 | MH586662 |
| 95 | 534 | Gammaproteobacteria | *Pseudoxanthomonas japonensis* | 100 | 534/534 | MH586663 |
| 96 | 548 | Gammaproteobacteria | *Pseudoxanthomonas mexicana* | 100 | 548/548 | MH586664 |
| 97 | 437 | Gammaproteobacteria | *Psychrobacter muriicola* | 100 | 437/437 | MH586665 |
| 98 | 553 | Gammaproteobacteria | *Psychrobacter pacificensis* | 100 | 553/553 | MH586666 |
| 99 | 542 | Gammaproteobacteria | *Psychrobacter piscatorii* | 99 | 544/545 | MH586667 |
| 100 | 521 | Alphaproteobacteria | *Rhizobium alkalisoli* | 100 | 521/521 | MH586668 |
| 101 | 450 | Alphaproteobacteria | *Rhizobium loessense* | 99 | 456/459 | MH586669 |
| 102 | 534 | Actinobacteria | *Rhodococcus aetherivorans* | 100 | 534/534 | MH586670 |
| 103 | 554 | Actinobacteria | *Rhodococcus agglutinans* | 100 | 554/554 | MH586671 |
| 104 | 547 | Actinobacteria | *Rhodococcus erythropolis* | 100 | 547/547 | MH586672 |
| 105 | 520 | Actinobacteria | *Rhodococcus globerulus* | 100 | 520/520 | MH586673 |
| 106 | 530 | Actinobacteria | *Rhodococcus jostii* | 99 | 534/536 | MH586674 |
| 107 | 537 | Actinobacteria | *Rhodococcus pedocola* | 100 | 537/537 | MH586675 |
| 108 | 535 | Actinobacteria | *Rhodococcus ruber* | 100 | 535/535 | MH586676 |
| 109 | 489 | Actinobacteria | *Rhodococcus triatomae* | 99 | 499/504 | MH586677 |
| 110 | 535 | Actinobacteria | *Rhodococcus tukisamuensis* | 100 | 535/535 | MH586678 |
| 111 | 522 | Alphaproteobacteria | *Rhodopseudomonas pseudopalustris* | 100 | 522/522 | MH586679 |
| 112 | 532 | Alphaproteobacteria | *Roseomonas aestuarii* | 100 | 532/532 | MH586680 |
| 113 | 467 | Alphaproteobacteria | *Roseomonas sediminicola* | 100 | 467/467 | MH586681 |
| 114 | 539 | Actinobacteria | *Saccharomonospora azurea* | 100 | 539/539 | MH586682 |
| 115 | 513 | Actinobacteria | *Saccharothrix ecbatanensis* | 100 | 513/513 | MH586683 |
| 116 | 516 | Alphaproteobacteria | *Sagittula stellata* | 99 | 524/528 | MH586684 |
| 117 | 501 | Bacilli | *Salinicoccus alkaliphilus* | 100 | 501/501 | MH586685 |
| 118 | 536 | Bacilli | *Salinicoccus hispanicus* | 100 | 536/536 | MH586686 |
| 119 | 526 | Actinobacteria | *Sinomonas halotolerans* | 99 | 530/532 | MH586687 |
| 120 | 541 | Alphaproteobacteria | *Sphingobium quisquiliarum* | 100 | 541/541 | MH586688 |
| 121 | 531 | Alphaproteobacteria | *Sphingomonas kyeonggiensis* | 100 | 531/531 | MH586689 |
| 122 | 534 | Alphaproteobacteria | *Sphingopyxis fribergensis* | 100 | 534/534 | MH586690 |
| 123 | 548 | Alphaproteobacteria | *Sphingopyxis italica* | 100 | 548/548 | MH586691 |
| 124 | 463 | Actinobacteria | *Streptomyces andamanensis* | 100 | 463/463 | MH586692 |
| 125 | 518 | Actinobacteria | *Streptomyces atrovirens* | 100 | 518/518 | MH586693 |
| 126 | 536 | Actinobacteria | *Streptomyces bambusae* | 100 | 536/536 | MH586694 |
| 127 | 521 | Actinobacteria | *Streptomyces bangladeshensis* | 100 | 521/521 | MH586695 |
| 128 | 536 | Actinobacteria | *Streptomyces coelicolor* | 100 | 536/536 | MH586696 |
| 129 | 525 | Actinobacteria | *Streptomyces griseoflavus* | 100 | 525/525 | MH586697 |
| 130 | 536 | Actinobacteria | *Streptomyces lateritius* | 100 | 536/536 | MH586698 |
| 131 | 514 | Actinobacteria | *Streptomyces leeuwenhoekii* | 100 | 514/514 | MH586699 |
| 132 | 535 | Actinobacteria | *Streptomyces ossamyceticus* | 100 | 535/535 | MH586700 |
| 133 | 535 | Actinobacteria | *Streptomyces pluripotens* | 100 | 535/535 | MH586701 |
| 134 | 484 | Actinobacteria | *Streptomyces polymachus* | 100 | 484/484 | MH586702 |
| 135 | 538 | Actinobacteria | *Streptomyces prasinus* | 100 | 538/538 | MH586703 |
| 136 | 538 | Actinobacteria | *Streptomyces pseudogriseolus* | 100 | 538/538 | MH586704 |
| 137 | 486 | Actinobacteria | *Streptomyces racemochromogenes* | 100 | 486/486 | MH586705 |
| 138 | 537 | Actinobacteria | *Streptomyces scopiformis* | 100 | 537/537 | MH586706 |
| 139 | 525 | Actinobacteria | *Streptomyces thermospinosisporus* | 100 | 525/525 | MH586707 |
| 140 | 494 | Actinobacteria | *Streptomyces toxytricini* | 100 | 494/494 | MH586708 |
| 141 | 538 | Actinobacteria | *Streptomyces wuyuanensis* | 100 | 538/538 | MH586709 |
| 142 | 535 | Actinobacteria | *Streptomyces yaanensis* | 100 | 535/535 | MH586710 |
| 143 | 517 | Actinobacteria | *Streptosporangium sandarakinum* | 99 | 527/531 | MH586711 |
| 144 | 530 | Actinobacteria | *Tessaracoccus oleiagri* | 99 | 536/539 | MH586712 |
| 145 | 518 | Alphaproteobacteria | *Tistrella bauzanensis* | 100 | 518/518 | MH586713 |
| 146 | 530 | Alphaproteobacteria | *Tistrella mobilis* | 100 | 530/530 | MH586714 |
| 147 | 535 | Actinobacteria | *Williamsia marianensis* | 100 | 535/535 | MH586715 |
| 148 | 540 | Alphaproteobacteria | *Xanthobacter flavus* | 100 | 540/540 | MH586716 |
| 149 | 533 | Alphaproteobacteria | *Zavarzinia compransoris* | 100 | 533/533 | MH586717 |


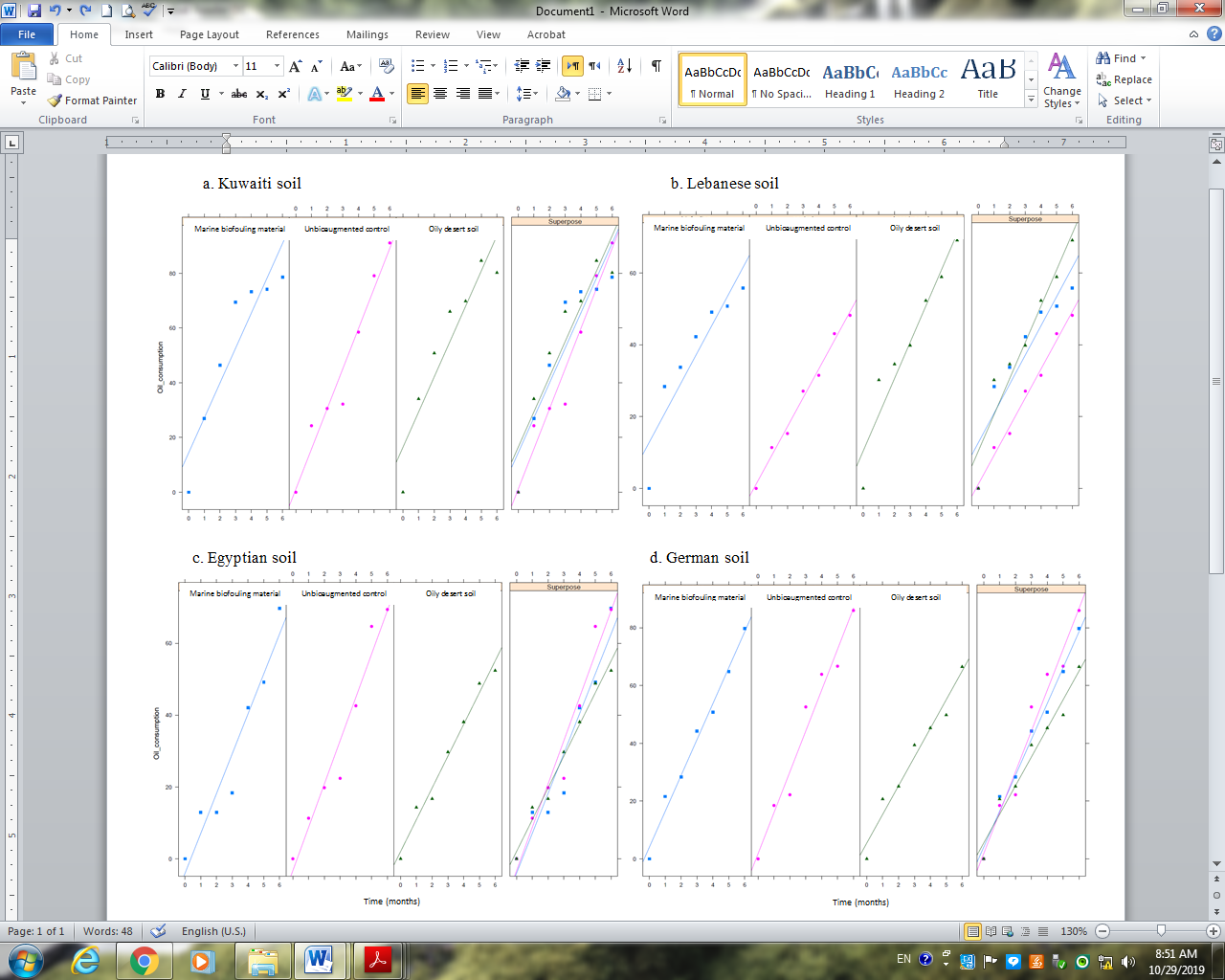


**Figure S1. Oil-consumption through 6 months in the four studied soils**

**
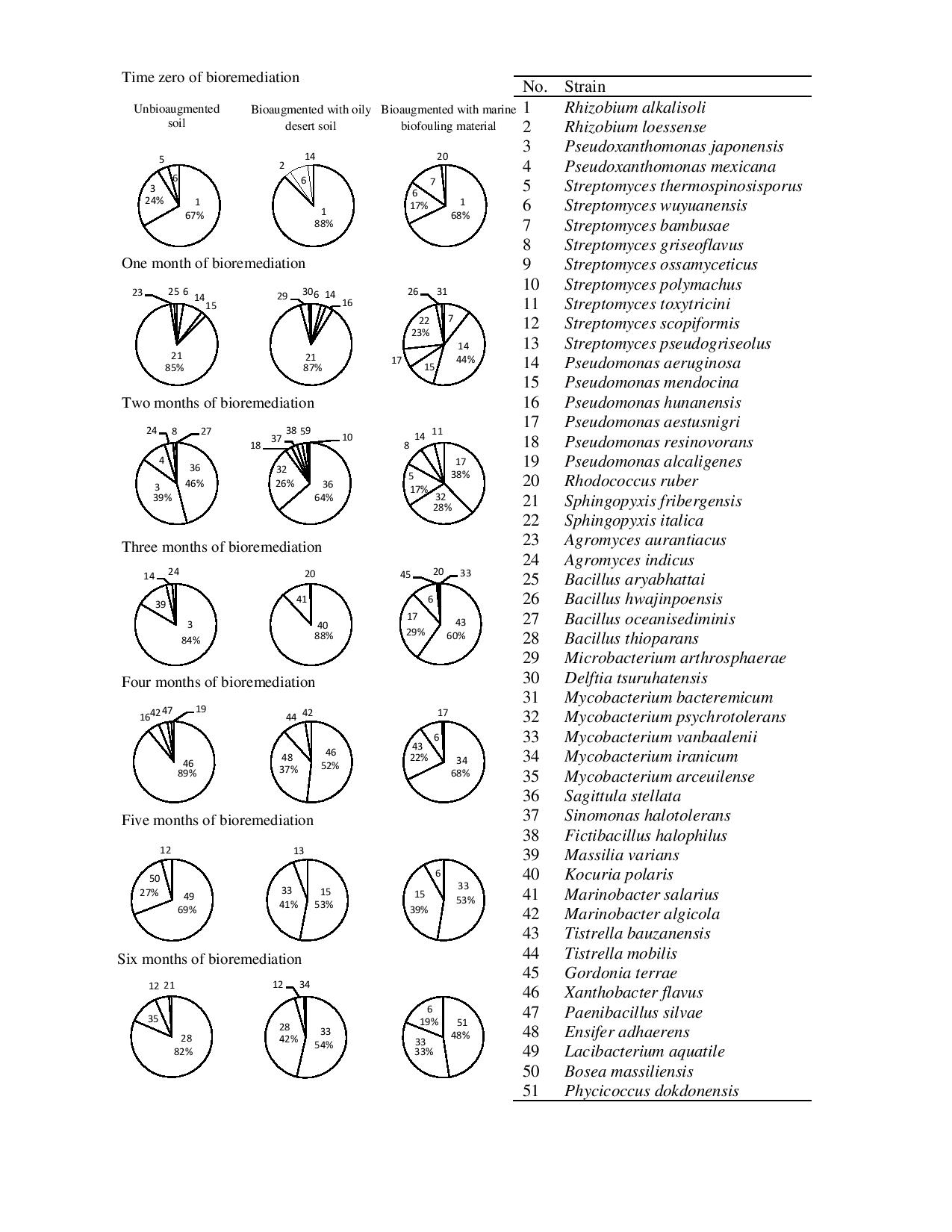
**

**Figure S2. Dynamics of hydrocarbonoclastic bacterial communities in oily soil from Kuwait during bench-scale bioremediation**

**
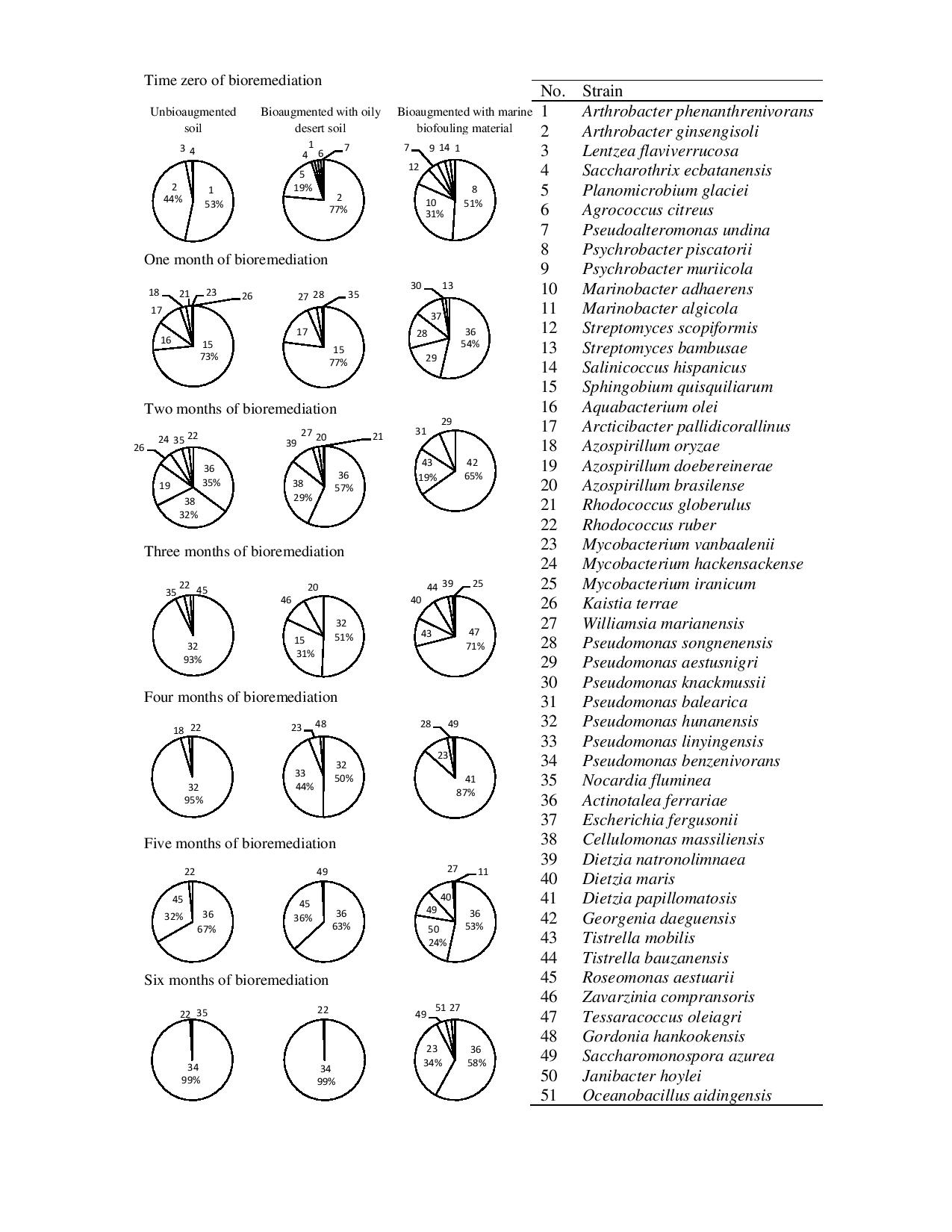
**

**Figure S3. Dynamics of hydrocarbonoclastic bacterial communities in oily soil from Lebanon during bench-scale bioremediation**

**
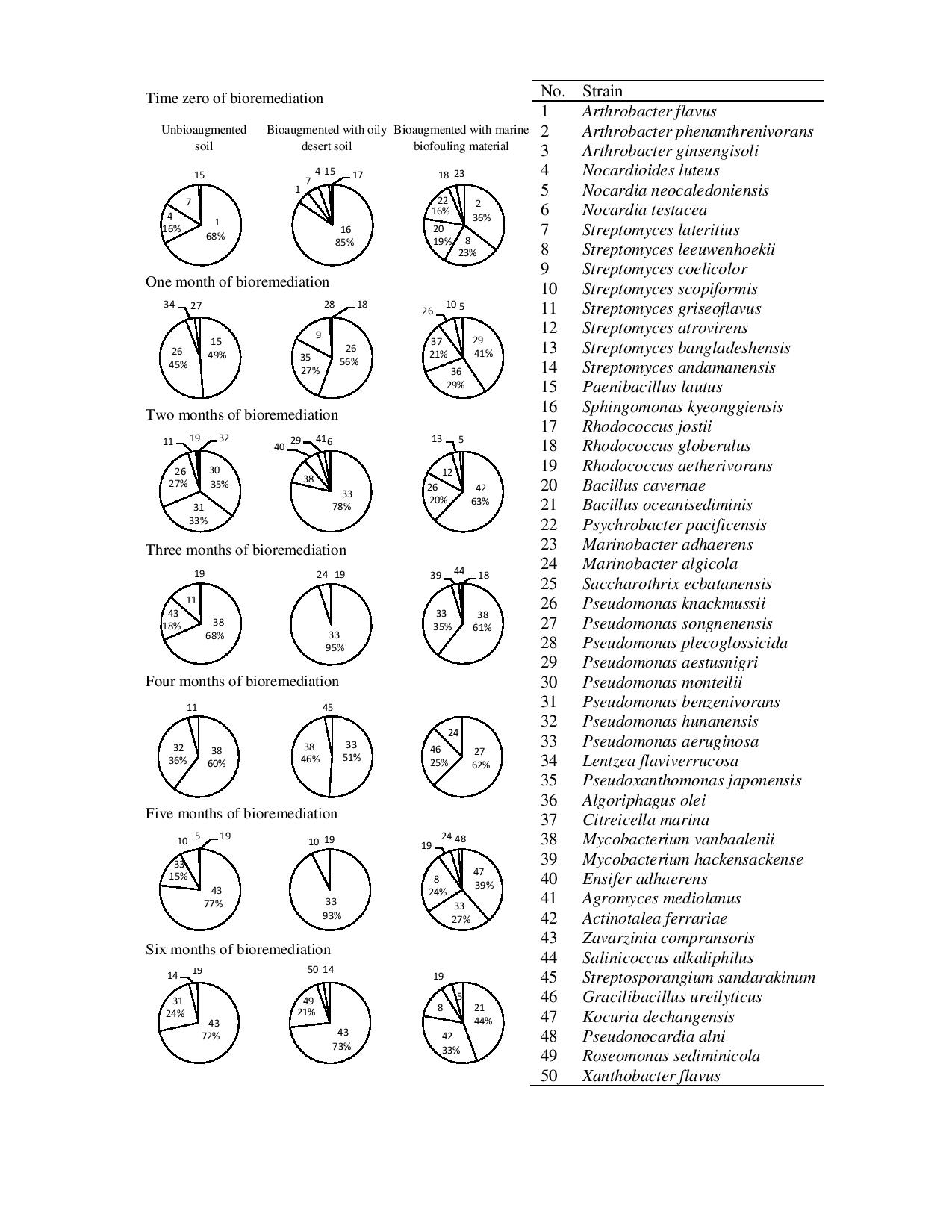
**

**Figure S4. Dynamics of hydrocarbonoclastic bacterial communities in oily soil from Egypt during bench-scale bioremediation**

**
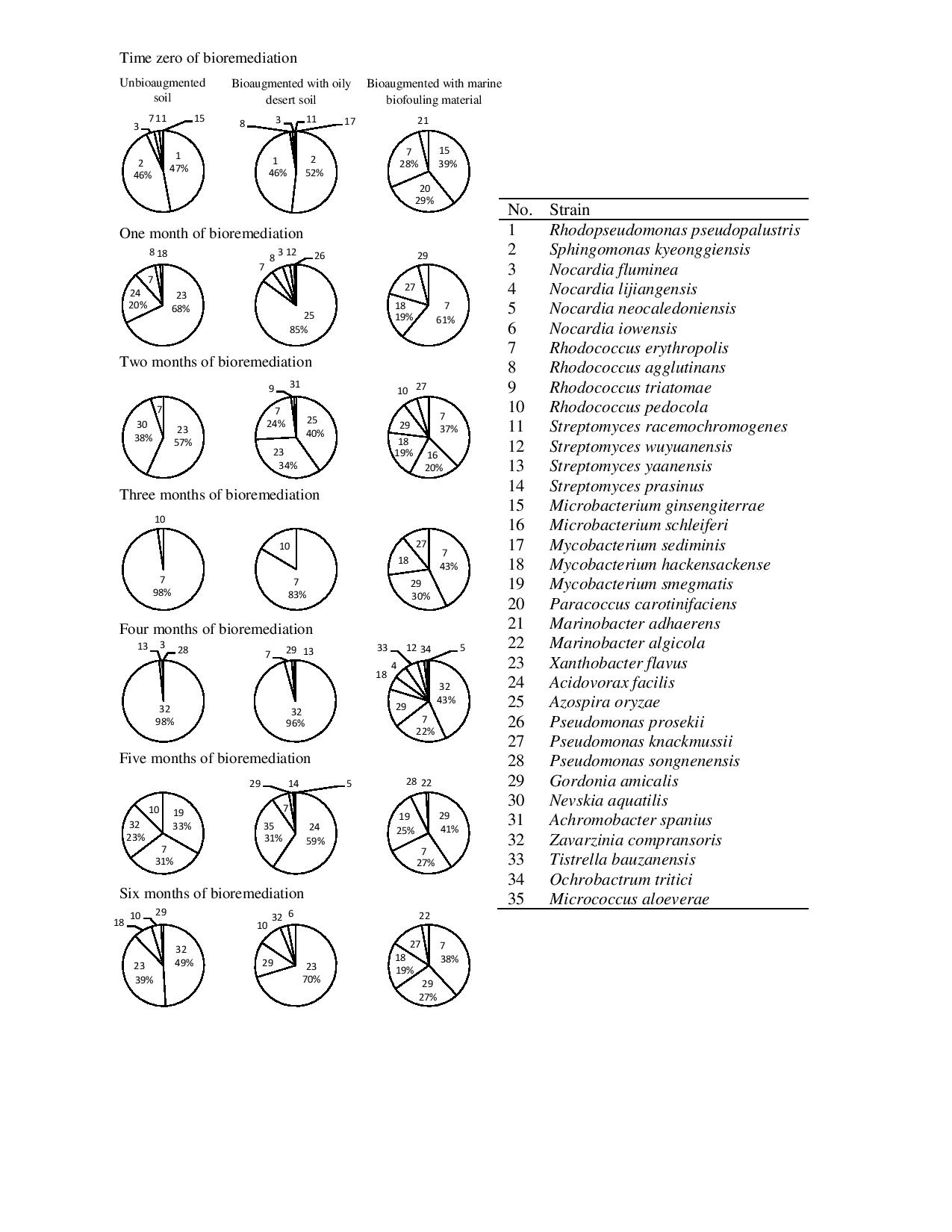
**

**Figure S5. Dynamics of hydrocarbonoclastic bacterial communities in oily soil from Germany during bench-scale bioremediation**


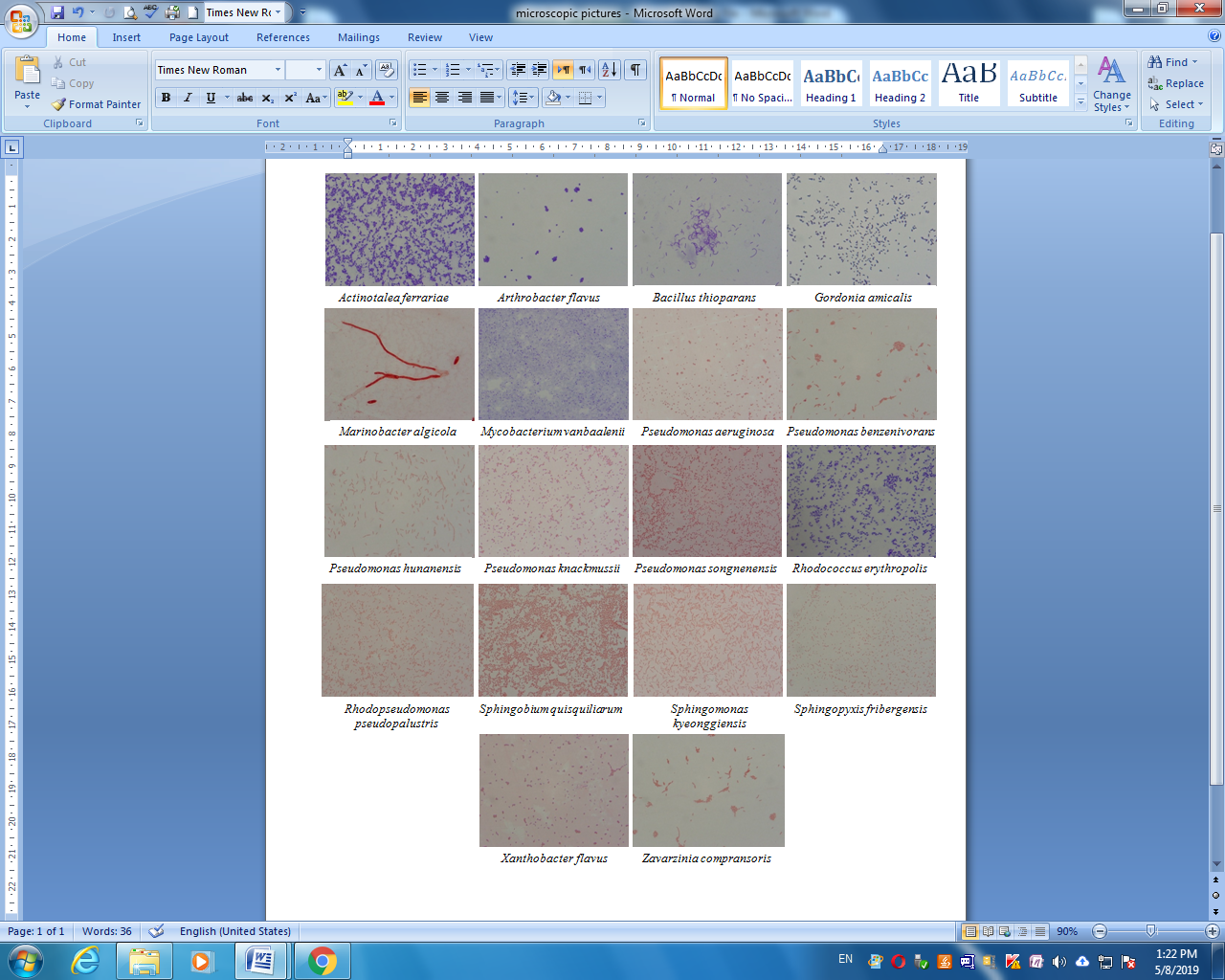


**Figure S6. Microscopic graph of 18 representative, predominant bacterial strains**
